# Cohort study of the association of antibody levels to AMA1, MSP1_19_, MSP3 and GLURP with protection from clinical malaria in Ghanaian children

**DOI:** 10.1186/1475-2875-7-142

**Published:** 2008-07-29

**Authors:** Daniel Dodoo, Anastasia Aikins, Kwadwo Asamoah Kusi, Helena Lamptey, Ed Remarque, Paul Milligan, Samuel Bosomprah, Roma Chilengi, Yaa Difie Osei, Bartholomew Dicky Akanmori, Michael Theisen

**Affiliations:** 1 Noguchi Memorial Institute for Medical Research, University of Ghana, Legon, Ghana; 2 Department of Parasitology Biomedical Primate Research Centre Lange Kleiweg, 139 2288 GJ, Rijswijk, The Netherlands; 3 Department of Infectious and Tropical Diseases, London School of Hygiene and Tropical Medicine, Keppel Street, London, WC1E 7HT, UK; 4 Ministry of Health, P.O. Box M44, Accra, Ghana; 5 The African Malaria Network Trust, Tanzania Commission for Science and Technology Building, P.O. Box 33207, Dar es Salaam, Tanzania; 6 Department of Biochemistry, University of Ghana, Legon, Ghana; 7 Department of Infectious Disease Immunology, State Serum Institute, Copenhagen, Denmark

## Abstract

**Background:**

Antigen-specific antibody-mediated immune responses play an important role in natural protection against clinical malaria, but conflicting estimates of this association have emerged from immuno-epidemiological studies in different geographical settings. This study was aimed at assessing in a standardized manner the relationship between the antibody responses to four malaria vaccine candidate antigens and protection from clinical malaria, in a cohort of Ghanaian children.

**Methods:**

Standardized ELISA protocols were used to measure isotype and IgG subclass levels to Apical Membrane Antigen 1 (AMA1), Merozoite Surface Protein 1–19 (MSP1_19_), Merozoite Surface Protein 3 (MSP3) and Glutamate Rich Protein (GLURP) antigens in plasma samples from 352 Ghanaian children, aged three to 10 years with subsequent malaria surveillance for nine months. This is one of a series of studies in different epidemiological settings using the same standardized ELISA protocols to permit comparisons of results from different laboratories.

**Results:**

The incidence rate of malaria was 0.35 episodes per child per year. Isotype and IgG subclasses for all antigens investigated increased with age, while the risk of malaria decreased with age. After adjusting for age, higher levels of IgG to GLURP, MSP1_19_, MSP3 and IgM to MSP1_19_, MSP3 and AMA1 were associated with decreased malaria incidence. Of the IgG subclasses, only IgG1 to MSP1_19 _was associated with reduced incidence of clinical malaria. A previous study in the same location failed to find an association of antibodies to MSP1_19 _with clinical malaria. The disagreement may be due to differences in reagents, ELISA and analytical procedures used in the two studies. When IgG, IgM and IgG subclass levels for all four antigens were included in a combined model, only IgG1 [(0.80 (0.67–0.97), p = 0.018)] and IgM [(0.48 (0.32–0.72), p < 0.001)] to MSP1_19 _were independently associated with protection from malaria.

**Conclusion:**

Using standardized procedures, the study has confirmed the importance of antibodies to MSP1_19 _in reducing the risk of clinical malaria in Ghanaian children, thus substantiating its potential as a malaria vaccine candidate.

## Background

Malaria remains one of the most important causes of morbidity and mortality in the world. Current methods of control are only partially effective and, therefore, the development of a vaccine which can provide a high degree of protection is a priority. Antibody-mediated immune responses to malaria antigens are known to be involved in protecting against disease [1-4], but the antigens that induce protective antibodies have not been conclusively identified. Immuno-epidemiological studies from different laboratories have sometimes yielded conflicting results [5-8]. This may be partly due to differences in malaria endemicity and the use of different study designs, reagents, assay protocols and statistical methodologies. In an attempt to make such studies more comparable, the Afro-Immuno Assay (AIA) network project was initiated. The network includes six African Institutions in Gabon, Ghana, Burkina Faso, Senegal, Tanzania, and Zimbabwe and three European Institutions from Denmark, The Netherlands and France. The Afro-Immuno Assay network has developed standardized enzyme immuno assays [9-11] that ensure the use of the same reagents, protocols and statistical methods to assess the association between acquisition of malaria specific antibody responses to four potential malaria vaccine candidate antigens and possible protection from clinical malaria. Samples for the AIA multi-center project were retrospectively obtained from cohort studies in six different geographical and epidemiological settings, comprising low endemic to holoendemic areas. These antigens include the Glutamate Rich Protein (GLURP), the Merozoite Surface Protein 3 (MSP3) [12], the 19-kilo Dalton region of the Merozoite Surface Protein 1 (MSP1_19) _[13] and the Apical Membrane Antigen 1 (AMA1) [14], which are all thought to induce protective antibody responses through various mechanisms [15-18]. Vaccines incorporating these antigens are currently in clinical trials and are described in detail elsewhere [7,19-26]. It is likely that a future malaria vaccine will comprise multiple rather than single antigens and it is, therefore, useful to study natural immune responses to multiple malaria antigens in relation to incidence of malaria in a more standardized way. In this study, the standardized AIA ELISA procedures [9-11], were used to assess the relationship between incidence of clinical malaria and naturally acquired isotype and IgG subclass responses to these four leading malaria vaccine candidate antigens, AMA1, MSP1_19_, MSP3 and GLURP in Ghanaian children from three to 10 years of age.

## Materials and methods

### Study area, study population and morbidity surveillance

Samples used in this study were obtained in March 2002 from a longitudinal study conducted in Dodowa, in which 352 children aged three to 10 years (in the active phase of acquiring immunity to malaria), were enrolled in a study, whose original aim had been to assess the role of cytokine regulation and immunity to malaria. Dodowa is a semi-rural town in the Dangme West District of the Greater Accra Region of Ghana, about 50 km from the capital Accra and is an area of moderate and stable malaria transmission with a seasonal peak. Bed net coverage in this area was low, about 10% [27]. The study was approved by the Noguchi Memorial Institute's Ethical Review Board. After obtaining consent from parents, blood was obtained from each child and plasma stored at -20°C until use. Malaria episodes were detected using both active and passive surveillance implemented over a period of nine-months, spanning the entire malaria transmission season. Clinical and parasitological information was captured using a standard questionnaire. Each child was visited once a week and the child and the parents or guardians were asked about symptoms of malaria since the last visit and whether she/he had received any anti-malarial treatment. The child was then given a physical examination and the body temperature measured. Children with a history of fever within 48 hours and/or axillary temperature equal to or above 37.5°C had a rapid test for malaria parasitaemia using OptiMAL™ (DiaMed, FLOW Inc. Portland, Oregon) and then thick and thin blood films were prepared for microscopy for estimating the parasite density which was used later in the more specific malaria case definition employed for data analysis. Only children with measured or reported fever, and with a positive rapid test were treated with chloroquine (in accordance with the then prevailing national malaria treatment policy) and in the case of severe symptoms, the child was referred to the hospital. At monthly intervals, blood smears from finger pricks were obtained from all children irrespective of symptoms to estimate the prevalence of asymptomatic malaria infections. The parents of the children were instructed to report to the field team if the child had any symptoms of disease at any time. The qualifying case definition for malaria in the data analysis was reported fever and/or a measured temperature equal to or above 37.5°C, with parasitaemia ≥ 5,000 parasites/μl of blood; this case definition has been found to have 90% sensitivity and specificity in the study area [28-30]. Positive and negative control plasma used in ELISA measurements were obtained from adult Liberians and Danes respectively.

### Malaria antigens

AMA1 was from the *Pichia pastoris *expressed ectodomain of *Plasmodium falciparum *FVO strain comprising amino acids 25–545 [31] (Donated by A Thomas, Biomedical Primate Research Centre). GLURP was an *Escherichia coli *recombinant protein containing the conserved non-repeat N-terminal region (amino acids 25–514) called R0 [32] (Donated by M. Theisen, Statens Serum Institut). MSP1_19 _was a *Baculovirus *antigen of the C-terminal region of the merozoite protein surface 1, produced in insect cells infected with a recombinant Baculovirus containing a synthetic G-C enriched PfMSP1 gene (Palo Alto allele), coding for 43 N-terminal MSP1 precursor residues and 16 amino acid residues upstream of the "classical" MSP-1_19 _(NIS---FCS) [33] (Donated by S. Longacre, Institut Pasteur). The MSP3 antigen used in this study was a long synthetic peptide called LR55 (amino acids 181 – 276) of the merozoite surface protein 3 [34] (Donated by M. Theisen, Statens Serum Institut). All the antigens were provided through the AIA Project.

### Enzyme-linked immunosorbent assay (ELISA)

Specific isotype and IgG subclass levels against GLURP, MSP1_19_, MSP3 and AMA1 were measured using indirect ELISA according to the AIA standard ELISA protocols [9-11]. All antigens tested were optimized and shown to be stable for at least three weeks, when antigen-coated plates and serum/plasma dilutions are refrigerated. The subclass specific reagents used were selected on the basis of low cross reactivities among themselves. To control for inter-assay and day-to-day variations in the standardized ELISA procedure, three-fold serial dilutions of reference standard reagents (IgG, IgM and IgG1 to IgG4) were directly coated on each ELISA plate (Maxisorp Nunc, Denmark) at a start concentration of 1,000 ng/ml (100 μl/well). OD values for the test samples were converted into antibody units with the standard reference curves generated for each ELISA plate using a four parameter curve-fit Microsoft Excel-based application. Samples were re-tested if the coefficient of variation between duplicate absorbance values were higher than 15% and plates were also re-tested if the R-square value of the standard curve was less than 97%. The reference standards, PBS buffer blank, positive and negative control plasma pools that were included in each ELISA test plate allow for the determination of failed assay runs. The AIA ELISA procedure used in this study is described in detail elsewhere [10].

### Statistical analysis

Clinical data were double entered using Microsoft Fox Pro and immunological data using Excel. STATA version 9.2 (Statcorp, Texas) was used for statistical analysis. Children were considered to have a clinical malaria episode if they had parasitaemia of ≥ 5,000 parasites/μl, with a measured temperature ≥ 37.5°C or a history of fever in the last 48 hours [6,35,36].

For each antigen, Poisson regression was used to investigate the association between the levels of antibody measured at baseline and the incidence rate of the first (or only) episode of clinical malaria. The level of total IgG, IgM and each IgG subclass, were analysed for each antigen in turn. Antibody values were transformed to log base 2, so that the rate ratio represents the ratio of malaria incidence corresponding to a doubling of antibody level. To investigate whether the relationship between malaria incidence and antibody level was nonlinear, a likelihood ratio test was used to compare the fit of the model when antibody level was included as a categorical or a continuous variable. When there were zero antibody values, indicating levels below the detection limit, the zero (left censored) values were assigned a nominal value equal to half the smallest measured value for that variable. If the proportion of zero values was large, the variable was treated as categorical with the reference category containing the zero values and the positive values divided into three equal groups. A likelihood ratio test was used to determine the P-value for the association with malaria incidence. Age at enrolment was considered to be an important potential confounder, and was included in the regressions as a factor with categories defined by quintiles. To model seasonality in malaria incidence, the calendar month of surveillance was included in the models as a factor. To construct a parsimonious model using all the immunological variables, firstly a model was produced for each antigen; in this model each IgG subclass, total IgG and IgM were candidates for inclusion provided the P-value for association with malaria incidence was 0.1 or less when considered individually. Variables were then removed from the model if the P-value for the likelihood ratio test was more than 0.1, provided removal did not change coefficients of variables in the model by more than 10%. In a second stage, the variables included in these models were candidates for inclusion in a final model derived in a similar way. Baseline parasitaemia was not considered as a potential confounder, but the interaction between each immunological variable and the presence of parasitaemia at baseline in their effects on malaria incidence was examined. A consequence of using a more specific case definition in the analysis than was used to decide treatment during the study is that children could have received anti-malarial treatments during the period they are considered at risk in the analysis. To explore the impact of these drug treatments, a time dependent variable was defined, to allow for a reduced risk of malaria for a period of 28 days after each drug treatment, which was included as a covariate in the Poisson regression model. LOESS smoothing was applied in R software to plot antibody levels in relation to age. Spearman's rank correlation test was used to assess associations between antibody levels and age.

## Results

### Pattern of *P. falciparum *infections and malaria in the study cohort

**Figure 1:**
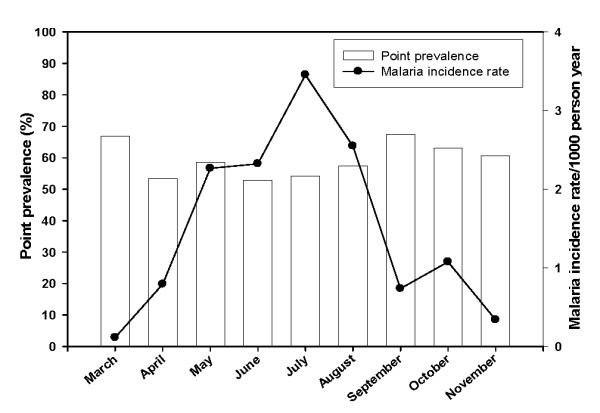
**The monthly point prevalence of asymptomatic malaria parasitaemia and incident rate of clinical malaria**. The point prevalence of asymptomatic parasitaemia for each month is represented as a bar graph and the pattern of clinical malaria (incident rate) is shown as a line graph.

Of the 352 children recruited for the original study, eight were lost to follow-up immediately after the baseline blood sampling. Of the 344 children followed up, sixty four (19%) had at least one episode of malaria (53 children had one episode, nine had two and two had three episodes). The incidence rate of malaria in the study cohort was 0.35 attacks per child per year (Table 1). The risk of clinical malaria decreased with increasing age [37]. Sixty-six percent of the children had asymptomatic parasitaemia at baseline. Parasites were predominantly *P. falciparum *(95%) and the prevalence of parasitaemia measured each month was roughly constant (ranging from 50% to 65%). The incidence of clinical malaria, however, varied during the survey period, rising gradually from March to May, peaking in July and then decreasing until November (Figure 1).

**Table 1:** Malaria incidence by age group

**Age in yrs:**	**No. of** ** children**	**Cum. incidence** ** of malaria**	**no episodes of malaria** ** (years at risk)**	**Incidence rate per ** **child year (95%CI)**
3	32	12/32 (38%)	15 (19.52)	0.79 (0.48–1.31)
4–5	86	24/86 (28%)	28 (54.75)	0.51 (0.35–0.74)
6–7	79	11/79 (14%)	13 (51.38)	0.25 (0.15–0.44)
8	48	6/48 (13%)	7 (31.11)	0.22 (0.11–0.47)
9–10	99	11/99 (11%)	14 (61.22)	0.23 (0.14–0.39)

Total	344	64/344 (19%)	77 (217.48)	0.35 (0.28–0.44)

### Relationship between age and antibody levels

**Figure 2:**
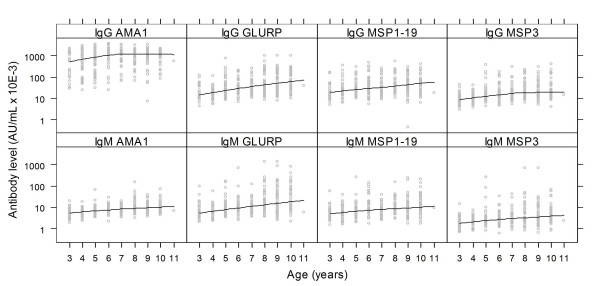
**Baseline IgG and IgM levels in relation to age**. The top and bottom panels represents total IgG or IgM levels to AMA1, GLURP, MSP1_19 _and MSP3 in relation to age of Ghanaian children, respectively. The line shows the LOESS smoothed estimate of the geometric mean.

**Figure 3:**
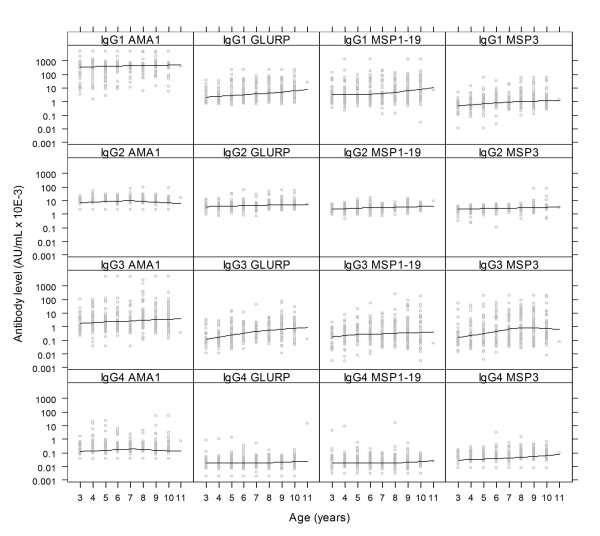
**Baseline IgG subclass levels in relation to age**. The top, 2nd, 3^rd ^and 4^th ^panels represent IgG subclasses 1 to 4 against AMA1, GLURP, MSP1_19 _and MSP3 in relation to age of Ghanaian children, respectively. The line shows the LOESS smoothed estimate of the geometric mean.

Several studies in malaria endemic regions have shown increasing antibody levels with age. This pattern is more pronounced the greater the intensity and duration of malaria transmission. In this study, the relationship between antibody levels and age were assessed for antibodies against MSP1_19_, AMA1, GLURP and MSP3. The levels of IgG and IgM to MSP1_19_, MSP3, AMA1 and GLURP increased with age (Spearman correlation coefficient (r_s_) 0.21 – 0.45; p < 0.001, Figure 2). IgG levels to AMA1 however, gradually increased until six years of age, and then leveled off. For the four antigens tested, IgG1, IgG2 and IgG3 significantly increased with age (r_s_, 0.12 – 0.36; p < 0.03) with the exception of IgG2 to AMA1. There was no evidence that the level of IgG4 was associated with age for any of the antigens. Like IgG to AMA1, IgG3 to MSP3 steadily increased with age until seven years of age, then leveled off (Figure 3).

The levels of both IgG and IgG1 were highest for AMA1 and lowest for MSP3, whereas IgG levels to MSP1_19 _and GLURP were comparable (Figures 2 and 3). The levels of IgG3 were higher in AMA1 than the comparable levels in GLURP, MSP1_19 _and MSP3 (Figure 3). In general, the levels of cytophilic IgG1 and IgG3 were higher than those of non-cytophilic IgG2 and IgG4, IgG4 levels being the lowest.

### Antibody levels in relation to protection from clinical malaria

Total IgG to MSP3, MSP1_19_, and GLURP, and IgM to all four antigens tested (MSP3, MSP1_19_, GLURP, and AMA1) were associated with reduced malaria incidence in crude analyses (Table 2). The incidence of both clinical malaria and antibody levels were associated with age, age is therefore a potential confounder, and it is important to adjust for its effects. After adjusting for the effect of age, there was evidence of a significant association between total IgG to MSP3, MSP1_19_, GLURP and reduced risk of malaria (IgG to MSP3: rate ratio 0.69 (95%CI 0.53, 0.90) P = 0.01; IgG to MSP1_19_: 0.75 (0.61, 0.92) P = 0.01; IgG to GLURP: 0.79 (0.64, 0.98) P = 0.04); and IgM levels to AMA1, MSP3, MSP1_19 _were also significantly associated with reduced risk of malaria (Table 2).

**Table 2:** Association of total IgG and IgM with malaria incidence

**IgG**	**Crude IRR (95%CI)**	**P-value**	**IRR adjusted for age (95%CI)**	**P-value**
MSP3	0.59 (0.45, 0.76)	< 0.0001	0.69 (0.53, 0.90)	0.01
MSP1_19_	0.74 (0.63, 0.88)	< 0.0001	0.75 (0.61, 0.92)	0.01
GLURP	0.68 (0.55, 0.82)	< 0.0001	0.79 (0.64, 0.98)	0.04
AMA1	0.96 (0.84, 1.09)	0.50	1.03 (0.89, 1.20)	0.67
**IgM**:				

MSP3	0.62 (0.49, 0.80)	< 0.0001	0.76 (0.59, 0.97)	0.03
MSP1	0.59 (0.47, 0.75)	< 0.0001	0.68 (0.53, 0.88)	< 0.01
GLURP	0.71 (0.58, 0.87)	< 0.01	0.84 (0.68, 1.03)	0.09
AMA1	0.48 (0.34, 0.67)	< 0.0001	0.63 (0.44, 0.91)	0.01

Malaria incidence rate ratios (IRR) with 95% confidence interval, corresponding to a doubling of baseline antibody level, before and after adjustment for the effect of age.

A large proportion of the measurements of IgG4 to MSP3 and AMA1 and IgG2 to AMA1 were zero (left-censored) values and so these variables were treated as categorical variables (Table 3). In the crude analysis, IgG1, IgG2 and IgG3 to MSP3, MSP1_19_, GLURP and also IgG4 to MSP3, were associated with a reduced risk of clinical malaria (Table 3, 4), but after adjustment for age, only one of these variables, IgG1 to MSP1_19_, remained significantly associated with malaria incidence (rate ratio 0.89 (95%CI 0.80, 0.99), P = 0.04, Table 4).

**Table 3:** Association of IgG2 and IgG4 with malaria incidence.

**Antigen**	**Antibody**		**Crude IRR (95%CI)**	**P-value**	**Age-adjusted IRR**	**P-value**
**MSP3**						

	IgG2*	(coded as log(2))	0.74 (0.57, 0.98)	0.04	0.85 (0.63, 1.15)	0.29
	IgG4	< detection limit	1	0.01	1	0.20
		< median	0.45 (0.21, 0.95)		0.56 (0.26, 1.19)	
		≥ median	0.47 (0.28, 0.79)		0.68 (0.40, 1.15)	
**MSP1**_19_						
	IgG2*	(coded as log(2))	0.62 (0.45, 0.86)	< 0.01	0.836 (0.606, 1.157)	0.275
	IgG4*	(coded as log(2))	1.07 (0.95, 1.21)	0.25	0.838 (0.608, 1.159)	0.278
**GLURP**						
	IgG2*	(coded as log(2))	0.65 (0.49, 0.86)	< 0.01	0.79 (0.58, 1.08)	0.13
	IgG4*	(coded as log(2))	0.94 (0.81, 1.08)	0.37	1.01 (0.87, 1.16)	0.94
**AMA1**						
	IgG2	< detection limit	1	0.18	1	0.54
		< median	1.80 (0.86, 3.78)			
		≥ median	1.78 (0.90, 3.51)			
	IgG4	< detection limit	1	0.19	1	0.54
		< median	0.96 (0.47, 1.93)			
		≥ median	0.62 (0.36, 1.07)			

For IgG 2 to AMA1 and for IgG4 to AMA1 and MSP3, antibody level was categorized.

*For IgG2 to MSP3, MSP1_19 _and GLURP, the association is expressed as the incidence rate ratio (IRR) corresponding to a doubling of baseline antibody level.

**Table 4:** Association of IgG1 and IgG3 with malaria incidence

**IgG1:**	**Crude IRR** ** (95%CI)**	**P-value**	**IRR adjusted for age** ** (95%CI)**	**P-value**
MSP3	0.82 (0.72, 0.94)	< 0.01	0.88 (0.76, 1.03)	0.11
MSP1_19_	0.86 (0.77, 0.97)	0.01	0.89 (0.80, 0.99)	0.04
GLURP	0.85 (0.74, 0.98)	0.02	0.93 (0.81, 1.08)	0.35
AMA1	0.95 (0.86, 1.06)	0.36	1.01 (0.90, 1.14)	0.83
**IgG3:**				

MSP3	0.90 (0.82, 0.98)	0.01	0.94 (0.86, 1.03)	0.17
MSP1_19_	0.90 (0.82, 0.99)	0.03	0.93 (0.84, 1.03)	0.18
GLURP	0.83 (0.74, 0.93)	< 0.01	0.91 (0.80,1.03)	0.14
AMA1	0.97 (0.89, 1.06)	0.48	0.98 (0.90, 1.07)	0.73

The incidence rate ratio (IRR) corresponds to a doubling of baseline antibody level, before and after adjustment for effects of age

When all immunological variables were considered simultaneously, only two variables were independently associated with reduced malaria incidence, IgG1 [(0.80 (0.66–0.96), p = 0.018)] to MSP1_19_, and IgM [(0.48 (0.32–0.72), p < 0.001)] to MSP1_19 _(Table 5).

**Table 5:** Adjusted rate ratios for immunological variables independently associated with malaria risk in the final model.

	**Crude IRR** ** (95%CI)**	**P-value**	**IRR adjusted for age** ** (95%CI)**	**P-value**	**IRR adjusted for effects of age** ** and treatment (95%CI)**
IgM to MSP1_19_	0.44 (0.30–0.64)	P < 0.001	0.49 (0.33–0.73)	P < 0.001	0.48 (0.32–0.72)
IgG1 to MSP1_19_	0.78 (0.65–0.93)	P = 0.005	0.80 (0.66–0.96)	P = 0.018	0.80 (0.67–0.97)
Treatment effect	0.28 (0.07–1.15)	P = 0.076	-	-	0.25 (0.06–1.05)

The estimated rate ratio for the effect of antimalarial treatments given to children with parasitaemia < 5000/uL was 0.25 (95%CI 0.06–1.05), indicating these children had a substantially reduced risk of being found positive with parasitaemia >= 5000/ul during the 28 days following the treatment. But the estimates of rate ratios for other variables in the model were unchanged when this variable was included in the model suggesting treatment effects did not cause a bias in the estimation of the effects of immunological variables.

## Discussion

This study in Ghanaian children is one of a series of studies designed to assess, using standardized methods, the association of antibody levels to four leading asexual blood-stage malaria antigens (MSP1_19_, MSP3, AMA1 and GLURP) with the incidence of clinical malaria in different epidemiological settings. Previous results have been difficult to interpret due to different study protocols and analytical methods having been used [5-7,9,14,29,38-40]. In this study, the prevalence of asymptomatic malaria parasitaemia was relatively high and stable, while incidence of clinical malaria fluctuated in parallel with the intensity of transmission and seasonal rainfall pattern. These patterns are typical of this area and have been reported in previous studies [6,41-43]. The variation in the incidence of clinical malaria during the study period may be due to the introduction of new parasites with different antigenic presentation into the population leading to clinical malaria in susceptible individuals. The risk of malaria decreased with age, while isotype and IgG subclass levels to the four antigens generally increased with age. This is consistent with the hypothesis that immunity to malaria is largely effected through antibody-mediated mechanisms and that protective antibody levels to relevant antigens increase with age-related exposure to the parasites [44]. Increasing IgG and IgM levels with age may reflect greater cumulative exposure of older children but may also be due to older children having a more mature immune system [45]. The association of IgM responses with reduced malaria incidence indicates a possible role in immunity in Ghanaian children. Although much emphasis has been placed on IgG as the important isotype in immunity against malaria, IgM, which has lower affinity but is multivalent, may afford protection via other mechanisms such as the blocking of merozoite invasion of erythrocytes, complement activation, agglutination of merozoites [46].

The association with malaria incidence of IgG responses to MSP3, MSP1_19 _and GLURP is consistent with data from several other immuno-epidemiological studies [5,7-9,12,29,30,38,47-49] indicating that these antigens may be targets of protective antibodies [1,2,4]. There was no evidence that IgG levels to AMA1 were associated with malaria incidence, and there was no evidence of an interaction with baseline parasitaemia in contrast with similar studies conducted in Kenya [14]. As shown in other studies, the cytophilic antibody levels to the four antigens tested in this study were higher than the non-cytophilic ones, emphasizing their importance in anti-malaria immunity [9,29,38,50,51]. IgG and cytophilic antibody levels were highest for AMA1, while the levels were relatively low for MSP3. These differences in specific antibody levels may be related to the number of immunogenic B-Cell epitopes exposed to the immune system and could also be related to the structure, location and function of the particular antigen(s). With the exception of IgG2 levels to AMA1, IgG2 levels to GLURP, MSP1_19 _and MSP3 increased with age which may suggest that IgG2 is involved in immunity against malaria. In recent studies, malaria antigen specific IgG2 have been shown to bind with high affinity to mutant Fcγ RII H131->R receptors [52] on monocytes, granulocytes and B cells, thus affording protection against malaria through monocytes and or neutrophil mediated mechanisms in subjects expressing the mutant CD32 form [53]. There was however, no evidence found that IgG2 was associated with malaria incidence for any of the four antigens tested.

The IgG subclasses, IgG1, IgG2 and IgG3 for MSP3, MSP1_19_, GLURP and IgG4 to MSP3 were associated with a reduced risk of malaria in un-adjusted analysis but of these only IgG1 to MSP1_19 _was independently associated with malaria incidence after adjustment for age. Other studies have shown the importance of IgG1 in clearing parasitaemia in children [5,47,54] In a previous cohort study conducted in the same area, there was no association between antibody levels to MSP1_19 _and malaria incidence [6]. This may be due to differences in antigen and antibody reagents used in the two studies; the MSP1_19 _used in this study was a Baculovirus product that included a synthetic G-C enriched PfMSP1 gene that coded for the 43 N-terminal MSP1 precursor residues and 16 amino acid residues upstream of the "classical" MSP-1_19 _(NIS---FCS) [33] compared to the one produced in *E. coli*, which had been used in the previous study. Although antibodies to MSP1_19 _have been shown to be associated with both exposure and protection from disease, the fine specificities of such responses may contribute to protection [40]. The antigen used in this study may have assessed antibodies of fine specificities that are protective [40,55,56], whereas the antigen used in the previous study did not. It may, therefore, be important to assess in a standardized way the various MSP1_19_, and other antigens, that are produced in different expression systems, in order to select the most appropriate antigen/expression system for malaria vaccine development. In another study in this series, in Burkina Faso, the same antigens, reagents, ELISA procedure and analytical methods were used; none of the isotypes and subclasses to MSP1_19 _was associated with the incidence of clinical malaria. Since the same laboratory methods were used, the different outcomes of these two studies may be attributed to differences in malaria transmission or to the age of the children [44]. In Burkina Faso, the malaria transmission season is much shorter, which may influence the induction of differing antibody types for controlling malaria as shown in recent studies conducted in areas with different malaria endemicities in Tanzania [9,44]. Although total IgG to GLURP and MSP3 were associated with the risk of malaria, none of the constituent subclasses was identified to be associated with protection. When the effects of all the immunological variables were considered simultaneously, only IgG1 and IgM to MSP1_19 _were independently associated with the incidence of clinical malaria, which may indicate the importance of MSP1_19 _in malaria vaccine development. Parasite growth inhibition assays would be required to confirm if this association reflects a functional role of MSP1_19 _in immunity.

## Conclusion

In conclusion, using standardized AIA ELISA, anti-MSP_19 _antibodies (IgG1 and IgM) have been shown to be the most strongly correlated with reduced risk of clinical malaria among the four malaria vaccine candidates tested. The standardized AIA ELISA developed for this project could be used to validate malaria vaccine candidate antigens, provide useful baseline information for clinical trials, and contribute to quality assured laboratory capacity in Africa.

## Authors' contributions

DD carried out field studies, developed assays and drafted the manuscript. AA performed the ELISA, compiled data and assisted in the manuscript writing. KAK and HL assisted with the ELISA. MT assisted with the assay development and manuscript writing, while PM, SB and ER wrote the analysis plan, performed the data analysis and together with RC, BDA and YDO contributed to the writing of the manuscript. All authors read and approved the final manuscript.
